# Enhanced Bio‐Electrochemical Reduction of Carbon Dioxide by Using Neutral Red as a Redox Mediator

**DOI:** 10.1002/cbic.201800784

**Published:** 2019-03-12

**Authors:** Hathaichanok Seelajaroen, Marianne Haberbauer, Christine Hemmelmair, Abdalaziz Aljabour, Liviu Mihai Dumitru, Achim Walter Hassel, Niyazi Serdar Sariciftci

**Affiliations:** ^1^ Linz Institute for Organic Solar Cells (LIOS) Institute of Physical Chemistry Johannes Kepler University Linz Altenberger Strasse 69 4040 Linz Austria; ^2^ The Austrian Centre of Industrial Biotechnology (acib GmbH) Stahlstrasse 14 4020 Linz Austria; ^3^ Institute of Chemical Technology of Inorganic Materials (TIM) Johannes Kepler University Linz Altenberger Strasse 69 4040 Linz Austria

**Keywords:** bioelectrocatalysis, CO_2_ reduction, direct electron injection, microbial electrosynthesis, redox mediator

## Abstract

Microbial electrosynthetic cells containing *Methylobacterium extorquens* were studied for the reduction of CO_2_ to formate by direct electron injection and redox mediator‐assisted approaches, with CO_2_ as the sole carbon source. The formation of a biofilm on a carbon felt (CF) electrode was achieved while applying a constant potential of −0.75 V versus Ag/AgCl under CO_2_‐saturated conditions. During the biofilm growth period, continuous H_2_ evolution was observed. The long‐term performance for CO_2_ reduction of the biofilm with and without neutral red as a redox mediator was studied by an applied potential of −0.75 V versus Ag/AgCl. The neutral red was introduced into the systems in two different ways: homogeneous (dissolved in solution) and heterogeneous (electropolymerized onto the working electrode). The heterogeneous approach was investigated in the microbial system, for the first time, where the CF working electrode was coated with poly(neutral red) by the oxidative electropolymerization thereof. The formation of poly(neutral red) was characterized by spectroscopic techniques. During long‐term electrolysis up to 17 weeks, the formation of formate was observed continuously with an average Faradaic efficiency of 4 %. With the contribution of neutral red, higher formate accumulation was observed. Moreover, the microbial electrosynthetic cell was characterized by means of electrochemical impedance spectroscopy to obtain more information on the CO_2_ reduction mechanism.

## Graphical abstract

**Three microbial electrosynthesis systems** containing *M. extorquens* are shown to reduce CO_2_ to formate by direct electron injection and redox mediator‐assisted (neutral red and poly(neutral red)) approaches. The enhancement in production rate and efficiency were observed in the systems containing neutral red. Long‐term electrolysis showed the continuous formation of formate with an average Faradaic efficiency of 4 %.

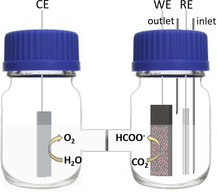

## Introduction

Over the past decades, atmospheric carbon dioxide (CO_2_) concentration has been increasing continuously and it is regarded as a major greenhouse gas.^1^, ^2^, ^3^ Thus, the reduction of atmospheric CO_2_ has attracted lots of interest as the carbon capture and utilization (CCU) processes.^4^ The CO_2_, as a carbon source, can be converted to several value‐added products,^5^, ^6^ such as carbon monoxide (CO), formic acids, acetic acid, methane, methanol and ethanol, through biological,^7^, ^8^, ^9^ electrochemical^10^, ^11^, ^12^, ^13^ and photo(electro)chemical approaches.^14^, ^15^, ^16^, ^17^, ^18^ However, CO_2_ conversion requires a large energy input due to the fact that CO_2_ is in a highly stable and low energy state. According to the thermodynamic reduction potentials, a one‐electron reaction from linear CO_2_ to its bent anionic form (CO_2_^⋅−^) requires a high potential of −1.90 V versus standard hydrogen electrode (SHE). Compared to the single‐electron process, the proton‐coupled multi‐electron reactions require substantially lower potentials.^16^ For example, the reduction potential of the CO_2_ reduction to formic acid (HCOOH) involving two electrons and two protons, is −0.61 V versus SHE.^16^ Nonetheless, the reduction potentials observed experimentally are much more negative than what thermodynamics predicts due to overpotentials. To overcome these high energy barriers, several approaches using catalysts were introduced.^19^, ^20^, ^21^, ^22^, ^23^, ^24^, ^25^, ^26^, ^27^

Among various CO_2_ reduction systems, the biological approach using microorganisms and enzymes as catalysts has been investigated due to the accessibility of catalysts from the biosphere compared to synthetically obtained catalysts. Moreover, biocatalysts provide high selectivity towards adducts and products and can be used under mild, ambient conditions without high temperature or high pressure.^28^, ^29^, ^30^ In the enzymatic approach, dehydrogenase enzymes have been reported as efficient catalysts for the CO_2_ conversion to alcohols, aldehydes and other hydrocarbons.^31^ Generally, these dehydrogenase enzymes only catalyze specific reactions with the requirement of a sacrificial cofactor.^31^, ^32^, ^33^ For example, formate dehydrogenase (FDH) is known to catalyze the reduction of CO_2_ to formate with the aid of nicotinamide adenine dinucleotide (NADH) as a cofactor.^34^, ^35^ Instead of using the sacrificial cofactors, the possibility of direct electron injection to dehydrogenase enzymes has been reported recently for electrochemical methods.^36^, ^37^, ^38^, ^39^ However, the main limitation of using enzymes is that the enzymes are isolated from specific microorganism strains and the purification processes require trained personnel and specific equipment.

In contrast to enzymes, living biocatalysts (microorganisms) provide a great advantage in terms of sustainable systems due to their ability to reproduce.^40^ Compared to FDH, which is very often sensitive to an oxygen‐containing environment, the structure of living microorganisms preserves the activity of enzymes inside due to their membrane and since they are able to reproduce themselves, new active enzymes are regularly generated. Additionally, the conversion efficiency of FDH for converting CO_2_ to formate is usually extremely low as compared to that for FDH converting formate to CO_2_.^41^ Several microorganisms have been already investigated in the field of CO_2_ reduction for their capability of capturing and/or converting CO_2_ to valuable products by using electrons from electron carriers, such as hydrogen (H_2_).^42^, ^43^, ^44^ In the 1930s, the first acetogenic bacteria (*Clostridium aceticum*) was investigated and the authors reported that CO_2_ and H_2_ could be converted to acetate, with the mechanism being described later on as the reductive acetyl‐CoA or the Wood–Ljungdahl pathway.^45^, ^46^, ^47^, ^48^ Moreover, the selectivity towards the products of CO_2_ reduction can be tuned corresponding to the local dehydrogenase enzymes of the microbial strains. Further investigation of these microorganisms reported that they are able to receive electrons either directly from the cathode or indirectly from the redox mediators of which the mechanism was called as extracellular electron transfer.^44^ Recently, Hwang et al. firstly reported the capability of *Methylobacterium extorquens*, which is known to be able to grow on reduced C1 compounds, to reduce CO_2_ to formate under aerobic conditions by using electrons supplied from an electrode through a mediator‐assisted approach.^49^, ^50^, ^51^

One possible approach to enhance the microbial electrosynthesis system is to introduce a redox mediator in order to facilitate the electron transfer from the electrode towards microorganisms’ cytoplasmic membrane. According to previous studies, 3‐amino‐7‐dimethylamino‐2‐methylphenazine hydrochloride (neutral red), known as a pH indicator and a staining dye, was used as an efficient redox mediator in several bio‐electrochemical systems because its redox potential is close to that of the NAD^+^/NADH redox couple, which is one of the major electron carrier in the microbial electron transport chain.^52^, ^53^, ^54^ Moreover, it is known that neutral red can be polymerized electrochemically on various substrates, especially carbon‐based ones, yielding a poly(neutral red) coating on the electrodes.^55^ As reported in previous studies, the resulting poly(neutral red) film was electrochemically active and chemically stable under biological conditions, and it has been widely used for bio‐electrochemical applications similar to those of the monomer, such as sensors^56^ and NAD^+^/NADH regeneration.^57^ From a practical point of view, a system containing a redox mediator coated or directly deposited onto the electrode is preferred, rather than the homogenous approach (a redox mediator dissolved in the electrolyte). This is firstly because product separation becomes easier and secondly because the electron transfer is greatly enhanced.^58^, ^59^, ^60^ Furthermore, such a system will have the advantage of long‐term operation and lower mediator costs.

In this study, we further investigated the long‐term performance of the microbial electrosynthetic cell (MEC) of *M. extorquens* by monitoring the products produced during the biofilm growth and CO_2_ reduction period. Additionally, the aid of neutral red as a redox mediator in an MEC system was investigated in two different approaches: homogeneous and heterogeneous ones. In the first approach studied, the cathode electrolyte contained soluble neutral red, whereas in the second approach the poly(neutral red) was directly coated onto the electrode.

## Results and Discussion

### Inoculation of microorganisms

**Figure 1:**
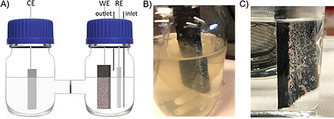
A) Schematic two‐compartment three‐electrode set‐up for microbial electrosynthesis experiments, and biofilm formation after one‐month inoculation on B) the CF electrode in the medium, and C) the PNR/CF electrode in 0.2 m phosphate buffer solution pH 7.0.

**Figure 2:**
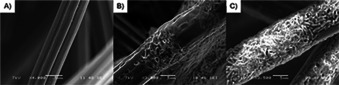
SEM images of A) bare CF, B) CF with biofilm and C) PNR/CF with biofilm. Scale bar: 5 μm.

*M. extorquens* biocathodes were developed in the two‐compartment electrochemical three‐electrode set‐up as shown in Figure 1 A. The cathodic compartment consisted of the medium containing 10 % (*v*/*v*) of *M. extorquens* pure culture suspension equipped with a carbon felt (CF) working electrode and a Ag/AgCl (3 m KCl) reference electrode. The anodic compartment contained a 0.2 m phosphate buffer solution at pH 7.0 equipped with a Pt plate counter electrode. A potential at −0.75 V was applied constantly for a month under CO_2_‐saturated atmosphere. The cathodic compartment was purged with CO_2_ approximately 2 h every week to keep the compartment saturated with CO_2_. Since *M. extorquens* is capable of extracellular electron transfer through their pili, it is able to take up the electrons directly from the electrode.^51^ Consequently, it can be inoculated electrochemically on a carbon‐based electrode. After a few days of inoculation, the growth of the biofilm on the electrode could be observed and increased gradually by the inoculation time. After one month of inoculation, the CF electrode was covered with the biofilm as shown in Figure 1 B. Additionally, the electrodes were dried under ambient conditions (pictures of electrodes are shown in Figure S1) overnight for optical microscope and SEM imaging as shown in Figure 2. The optical microscopic and SEM images show characteristic pink‐pigmented biofilm and rod‐shaped cells of methylobacterium, confirming the growth of *M. extorquens* biofilm.^62^, ^63^

**Figure 3:**
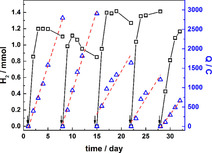
Amount of produced H_2_ (**□**) and electrical charge consumed (Q, **▵** ) during the inoculation period. The red dashed‐lines show linear fitting graph of electrical charge consumption. The arrows indicate when the cathodic chamber was purged with CO_2_.

During this period, headspace samples were collected daily and analyzed by gas chromatography (GC). The GC results showed that H_2_ was the only product detected in this phase. The amount of H_2_ produced is presented as a black line in Figure 3. The arrows indicate the period when the cathodic chamber was purged with CO_2_. The results reveal that H_2_ production was observed continuously and the highest H_2_ production was observed in the first day, immediately after CO_2_ purging with a production rate of 0.86–1.04 mmol/day. However, the lowering in H_2_ amount in the third to fifth day of the cycle might be because the microorganisms consumed H_2_ as a reducing agent in their metabolisms as described in the Wood–Ljungdahl pathway.^64^ Furthermore, the electrical charges (Q) consumed during this period were plotted together in Figure 3 (blue data points). These results showed that the electrons were consumed constantly during this period, not only in the region where H_2_ was produced. The red dashed lines were plotted in order to present the linear fitting of charge data points. The fitting provides the rates of electrical charges consumed in each purging cycle which were found to be in the rage of 170.5–418.8 C/day. The %FE of H_2_ production in the growing phase was calculated for one day immediately after CO_2_ purging. The %FEs were in the range of 35–98 %.

As the experiments were done in aqueous solution, H_2_ generation from electrochemical water‐splitting has to be taken into consideration. However, we believe that the H_2_ produced during the inoculation period is produced by microorganisms themselves, as part of their metabolism. To confirm this, control experiments were carried out by constantly applying the same potential of −0.75 V in the media by using a bare CF working electrode (without the biocatalyst). The experiments were done under N_2_‐ and CO_2_‐saturated conditions, and daily headspace samples were collected for a period of 1 week. The GC analysis revealed that no H_2_ was produced during the electrolysis of bare CF electrode in N_2_‐ or CO_2_‐saturated conditions (as shown in Figure S3 and S4). Considering the results obtained for these control experiments together with our observation that the production rate was increased after 2 weeks of inoculation related to the more biofilm formed on the electrode. Thus, we believe that the H_2_ constantly produced during the growth might be the product of microbial fermentation processes.^65^

In this work, we aimed to study the long‐term performance of the biocathodes resulting from the inoculation of *M. extorquens* and the effect of neutral red as a redox mediator both when the redox mediator is dissolved in the solution and when it was coated on the electrode. Three different electrosynthetic systems were designed as MEC 1, 2 and 3. MEC 1 is defined as the electrosynthesis system equipped with the biofilm CF working electrode. In the second cell (MEC 2), we investigated the effects of neutral red as a redox mediator in the solution. Therefore, the cell was equipped with the biofilm CF as a working electrode and buffer solution containing neutral red as the electrolyte solution. In addition, to study the effects of the redox mediator when it was directly attached on the electrode, MEC 3 was developed by using the biofilm coated on PNR/CF as a working electrode in mediator‐free buffer solution.

### Electropolymerization of neutral red

**Figure 4:**
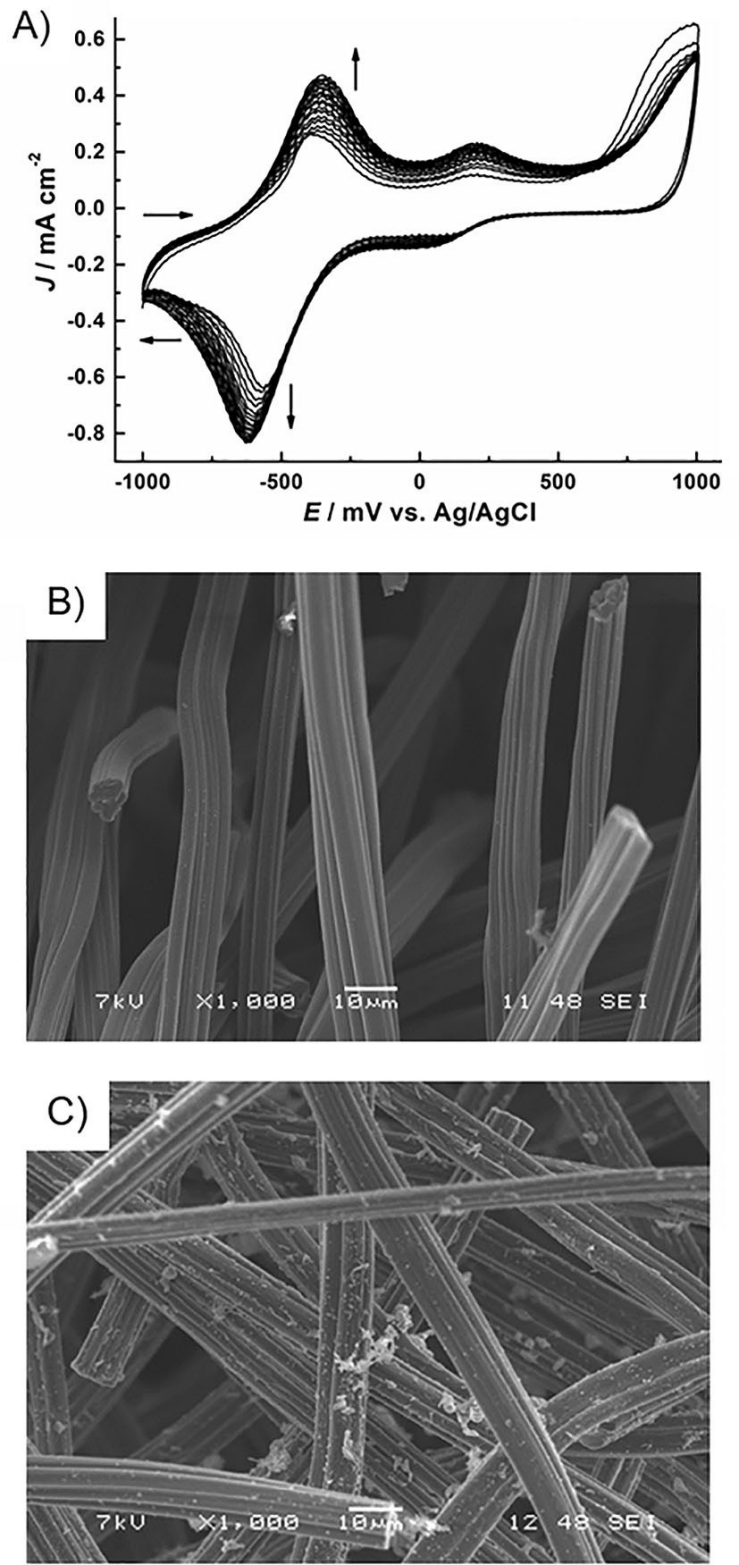
A) Cyclic voltammograms of neutral red electropolymerization onto a CF electrode: 1 mm neutral red in 0.1 m phosphate buffer solution pH 6.0 containing 0.1 m KNO_3_ at a scan rate of 50 mV s^−1^ over 20 cycles (arrows show the development of the cyclic voltammograms with cycles) and SEM images of B) a bare CF and C) PNR/CF. Scale bar: 10 μm.

The PNR/CF electrode was prepared by the oxidative electrochemical polymerization and used as working electrode in MEC 3. The electropolymerization was performed in 0.1 m phosphate buffer solution pH 6.0 containing 1 mm neutral red at potentials between −1.0 to 1.0 V with the scan rate of 50 mV s^−1^ over 20 cycles as shown in Figure 4 A, yielding a poly(neutral red)‐coating onto the CF electrode (PNR/CF). In the first cycle, three oxidation peaks at −0.39, 0.20 and around 0.8 V were observed. The first two redox couples indicated electrochemical features of protonated neutral red and its polymer, respectively. Another irreversible oxidation peak at around 0.8 V is referred to the formation of cation radical species that can initiate polymerization. These results are in agreement to the results reported previously.^55^, ^57^ Upon polymerization, the current of the first two redox couples increased as more redox active polymer is formed on the electrode. Furthermore, a positive shift in the potential positions and broader features were observed. These phenomena could be related to the changes in the electrode surface and the branching of the polymer. After removal of the remaining monomer solution by rinsing with water, the resulting PNR/CF electrode was electrochemically characterized by cyclic voltammetry. CVs were recorded in 0.1 m phosphate buffer solution pH 6.0 at different sweep rates (5, 10, 25, 50 and 100 mV s^−1^). Two redox couple peaks were observed corresponding to those of monomer peaks, showing the electrochemical monomer‐related features of the resulting film as shown in Figure S5. Furthermore, the SEM images of PNR/CF and bare CF were shown in Figure 4 B, revealing the formation of polymeric film.

**Figure 5:**
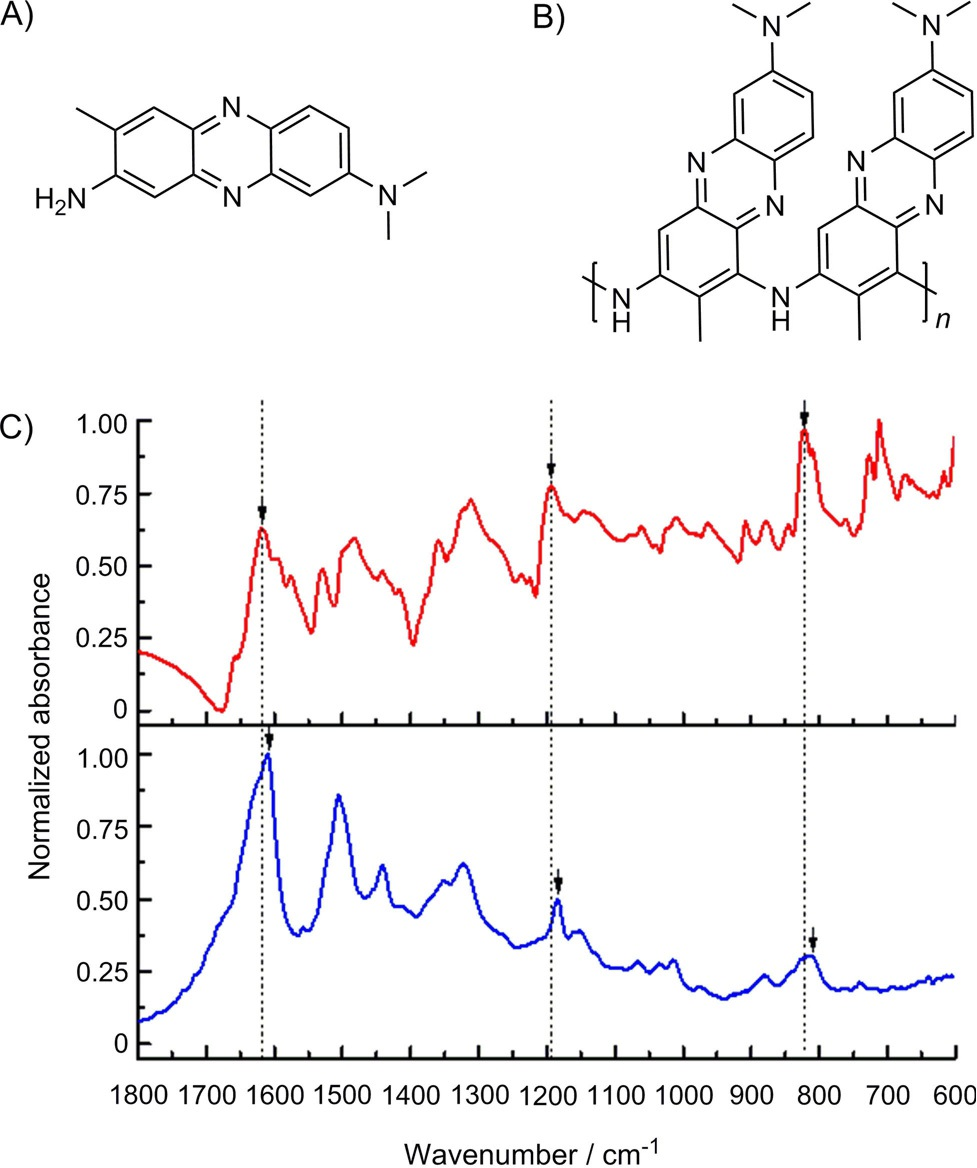
A) Structure of neutral red, B) structure of poly(neutral red) as proposed herein and C) FTIR spectra of neutral red ( **—** ) and poly(neutral red) ( **—** ).

With the similar manner to PNR/CF preparation, neutral red was electropolymerized onto Cr/Au‐coated glass electrodes for FTIR and UV/Vis measurements. Figure 5 shows FTIR spectra of neutral red (red line) and poly(neutral red) (blue line) together with the proposed structure of poly(neutral red). The FTIR spectrum of poly(neutral red) shows characteristic absorption peaks at 1609, 1185 and 812 cm^−1^, which are attributed to vibration of C=C or C=N, in‐plane bending of C−H and deformation of aromatic ring, respectively.^66^ The absorption peaks of poly(neutral red) were shifted 10 cm^−1^ for ν_C=C or C=N_, 7 cm^−1^ for *δ*_C−H_ and 9 cm^−1^ for aromatic ring deformation towards negative wavenumber, compared to those of neutral red monomer. The shifts referred to increase in π‐conjugation of the polymer. Additionally, UV/Vis absorption of the polymeric film was investigated, the spectra for which are shown in Figure S6. The absorption spectra exhibit maxima at wavelengths of 531, 500 and 455 nm for neutral red aqueous solution, drop‐casted neutral red monomer, and poly(neutral red) film, respectively. The absorption spectrum of poly(neutral red) film was consistent with its of monomer with broader features, which is attributed to higher aggregation of aromatic rings in the polymeric layers.

### Long‐term microbial electrosynthesis

After one‐month inoculation, the electrolyte solutions in both compartments were replaced completely with 0.2 m phosphate buffer solution pH 7.0. A constant potential at −0.75 V was applied under CO_2_‐saturated condition. In the cathode compartment, the clear buffer solution became partly cloudy because of the remaining medium absorbed in the CF electrode. Thus, the solution was replaced again with the buffer solution.

**Figure 6:**
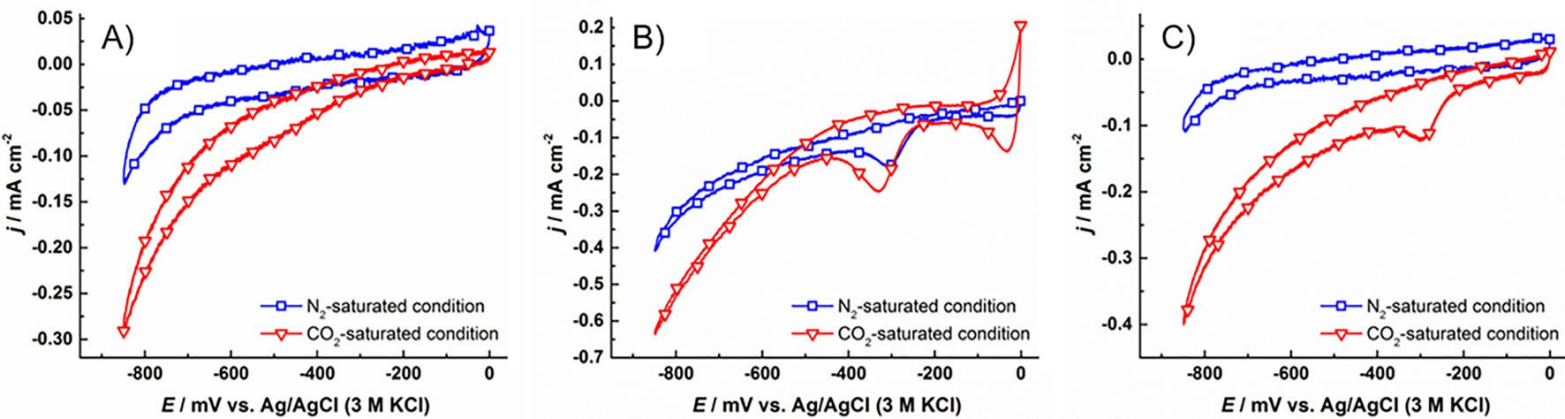
Cyclic voltammograms of biocathodes in A) MEC 1, B) MEC 2 and C) MEC 3 were recorded at potentials between 0 to −850 mV vs. Ag/AgCl (3 m KCl) with a scan rate of 1 mV s^−1^ in 0.2 m phosphate buffer solution pH 7.0 (with 50 μm of neutral red for MEC 2) saturated with N_2_ ( **□** ) and CO_2_ ( **▿** ).

In all MECs, the experiments were performed in the two‐compartment three‐electrode set‐up contained 0.2 m phosphate buffer solution equipped with the biofilm‐coated CF (or biofilm‐coated PNR/CF in MEC 3) working electrode, a Ag/AgCl (3 m KCl) reference electrode and a Pt plate counter electrode. The resulting biocathodes were characterized electrochemically by cyclic voltammetry under N_2_‐ and CO_2_‐saturated conditions and these results are shown in Figure 6 in blue and red lines, respectively. In all MECs, there is an increase in reductive current starting from −300 mV observed for CO_2_‐saturated systems as compared to N_2_‐saturated conditions indicating CO_2_ reduction as the predominant reaction. In the CVs of MEC 1, the reductive currents are lower compared to MEC 2 and MEC 3, showing enhancement in the electron‐transfer processes in the presence of neutral red as a redox mediator. The reductive peaks at around −300 mV in the CVs that were recorded in MEC 2 and 3 systems belonged to the reductive peaks of neutral red monomer. The observation of neutral red monomer peak in MEC 3 is assumed that it come from the degradation of poly(neutral red).

**Figure 7:**
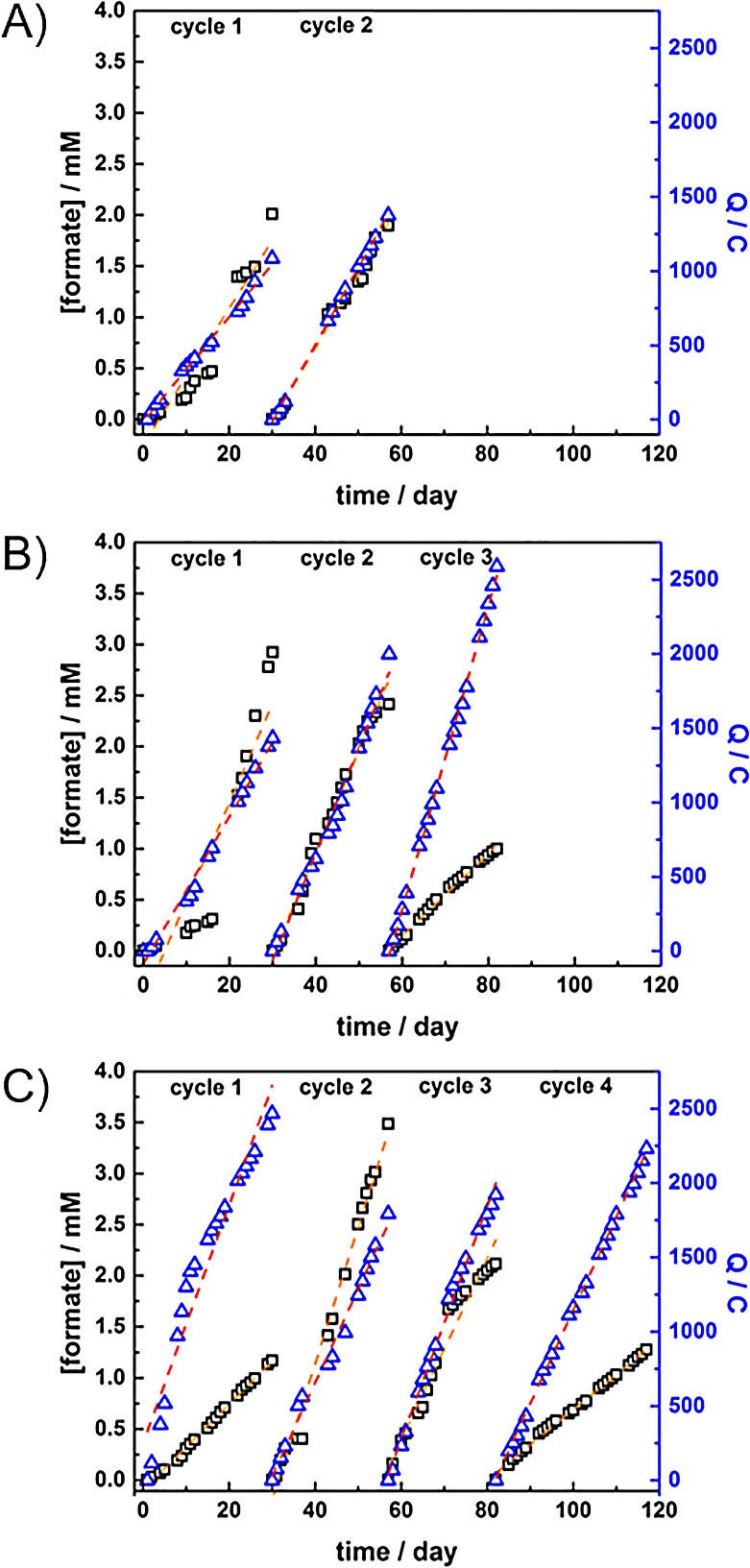
Plots of accumulated formate concentration (**□**), accumulated electrical charges (Q; **▵** ) of A) MEC 1 for 8 weeks, B) MEC 2 for 12 weeks and C) MEC 3 for 17 weeks. The dashed orange and red lines show linear fitting curve in each cycle of formate concentration and charges, respectively.

The long‐term performance studies of all MECs for the reduction of CO_2_ to formate were studied by applying a constant potential at −0.75 V under CO_2_‐saturated condition. The electrolysis was monitored by investigating the amount of formate produced in the solution using ion chromatography (IC) and the component of headspace gas using GC. The cathodic chamber was purged with CO_2_ for 2 h every 2 days to keep the system saturated with CO_2_ and the electrolyte solution was replaced every 3–4 weeks. MEC 1 was monitored over 8 weeks. In MEC 2, the experiment was performed in the presence of dissolved neutral red with a concentration of 50 μm in the cathodic electrolyte solution and was monitored over 12 weeks. Additionally, MEC 3 was developed and studied for 17 weeks. The running time of each MEC was limited to practical issues. According to the chromatography results, formate was the only product observed in all MECs. During the study, the electrolyte solution was replaced with a new solution every 3–4 weeks. Each electrolyte replacement was labelled sequentially, namely cycles 1–4. Figure 7 shows the plot of accumulative formate concentration and electrical charges consumed during each cycle and the results are summarized in Table 1. Moreover, the plots were linearly fitted (shown as dashed lines), resulting to the rates of formate production and charge consumption. In case of the overall running period, the estimated production rate was calculated from the total amount of produced formate and charges. In MEC 1, formate production was found to be moderately stable and reached 2.0 and 1.1 mm in cycles 1 and 2, with formate production rates of 66.7 and 71.7 μm/day, respectively. The corresponding %FEs were found to be 3 and 2 %. In MEC 2, the produced formate concentration reached 2.9, 2.4 and 1.0 mm in cycles 1, 2 and 3, respectively. The production rate increased significantly after the 16th running day in cycle 1, with the production rate of 96.9 μm day^−1^ for the first cycle. After that, the rate went down to 99.8 and 40.7 μm day^−1^ in cycles 2 and 3, respectively. While the current density of the system increased in every cycle. Consequently, calculated %FEs were substantially lower from 8 % in the first cycle, to 5 and 1 % in the second and third cycle, respectively. In case of MEC 3, formate was produced slowly in the first cycle with the rate of 42.1 μm day^−1^ and then reached the highest production rate of 133.5 μm day^−1^ in the second cycle. The rate decreased gradually to 88.8 and 34.4 μm day^−1^ in the third and fourth cycle, respectively. The current density remained stable and %FEs were found to be 2, 8, 4 and 2 %, in cycles 1, 2, 3 and 4, respectively. For comparison, another set of calculations was done for a 57‐day period. The results revealed that with the contribution of neutral red, 36 and 20 % more formate was accumulated in MEC 2 and MEC 3, respectively. However, we found out that after the second cycle, the systems containing neutral red were broken down, which might indicate that neutral red and poly(neutral red) degrade during the experiment. Compared to the previous report of the MEC‐containing methylobacterium, in which methyl viologen was used as an electron mediator, formate accumulated to reach a concentration of about 10 mm in 20 h.^51^ However, the cell reaction duration was only up to 80 h due to the unstable character of methyl viologen. Additionally, methyl viologen (also known as paraquat, a herbicide) is toxic to mammals therefore using methyl viologen is not desirable. Regarding these practical issues, neutral red systems are more promising.

**Table 1:** Comparison of total formate formation, accumulated charges, formate production rates, charge consumption rates and calculated average %FE in each cycle of three MECs.

System		Cycle 1 (30 days)	Cycle 2 (27 days)	Cycle 3 (25days)	Cycle 4 (31 days)	57 days	Overall
MEC 1	[formate] / mm	2.0	1.9	–	–	3.9	3.9
	Q / C	1086.4	1376.3	–	–	2462.7	2462.7
	production rate / μm day^−1^	66.7	71.9	–	–	68.6	68.6
	Q rate / C day^−1^	35.3	51.9	–	–	43.2	43.2
	%FE	3	2	–	–	2	2
MEC 2	[formate] / mm	2.9	2.4	1.0	–	5.3	6.3
	Q / C	1432.3	1997.6	2588.1	–	3429.9	6018.0
	production rate / μm day^−1^	96.9	99.8	40.7	–	93.7	77.3
	Q rate / C day^−1^	49.8	71.7	102.2	–	60.2	73.4
	%FE	8	5	1	–	6	4
MEC 3	[formate] / mm	1.2	3.5	2.1	1.3	4.7	8.1
	Q / C	2466.9	1793.7	1920.6	2231.2	4260.6	8412.5
	production rate / μm day^−1^	42.1	133.5	88.8	34.4	81.7	67.8
	Q rate / C day^−1^	80.1	63.0	78.3	62.9	74.7	71.9
	%FE	2	8	4	2	4	4

**Scheme 1:**
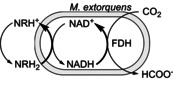
Proposed reaction mechanism at the electrode in the presence of neutral red. The NADH mechanism is intrinsically involved in living cells.

The mechanism of the electron transfer via neutral red mediator is not clear. It can be a redox mediator and/or a biological mediator. One of the proposed mechanisms of neutral red as a redox mediator in the homogeneous approach is presented in Scheme 1. When dissolved in neutral pH water, neutral red is in its acidic form (NRH^+^). While applying potential at −0.750 V versus Ag/AgCl (3 m KCl), NRH^+^ will be electrochemically reduced (NRH_2_; *E* ^0^=−0.535 V vs. Ag/AgCl (3 m KCl) for NRH_2_/NRH^+^).^54^ The resulting NRH_2_ diffuses into the inner cytoplasmic membrane and then is oxidized into NRH^+^ with the transfer of two electrons and one proton for the reduction of NAD^+^ to NADH (*E* ^0^=−0.530 V vs. Ag/AgCl (3 m KCl) for NAD^+^/NADH).^54^ NADH is known to be an essential coenzyme in the enzymatic reduction of CO_2_ to formate, catalyzed by formate dehydrogenase, and the reaction takes place inside the microbial cells. Consequently, with the presence of neutral red in the system, the electron transfer from the electrode to the reaction sites is greatly improved, thus higher production rates could be achieved. In addition, using immobilized redox mediators provides a practical system because the mediator is attached onto the electrode, thus, the problems of mediator contamination and product separation could be excluded. Additionally, PNR/CF was found out to be a suitable support for the microorganisms since better coverage of biofilm was observed on the electrode as shown in Figure 2 B and C, S1 and S2. It is attributed to higher nitrogen to carbon ratio from amine groups of poly(neutral red) coated onto the electrode which promote the bacteria adhesion to the electrode.^67^, ^68^, ^69^

Another set of control experiments were made in order to confirm that without bacteria, even when using neutral red, no formate production could be observed. The experiments were performed in a similar set‐up containing 0.2 m phosphate buffer solution pH 7.0 equipped with a CF electrode without microorganisms inoculated on the electrode. The first experiment was done by applying a constant potential of −0.75 V under N_2_‐saturated conditions for a week. After that, the cathode chamber was purged with CO_2_ for 2 h and the cell was monitored for an additional week. Then, neutral red solution was added into the cathodic solution and the experiment continued for another week. In all cases no formate production could be observed, confirming again that the CO_2_ reduction can only occur when the bio‐catalyst is present on the CF electrode (as shown in Figure S**7**).

To clarify the source of the carbon, another control experiment was done in MEC 2 by applying constant potential at −0.75 V under N_2_‐saturated atmosphere. The electrolysis was monitored by investigating the formate concentration and headspace sample analyzed with ion and gas chromatography, respectively. The results show that no formate product or any gas products was observed during the control experiments. This information confirmed that the observed formate was produced by *M. extorquens* inoculated on the CF electrode with CO_2_ as the carbon source in the system.

### Characterization by electrochemical impedance spectroscopy

**Figure 8:**
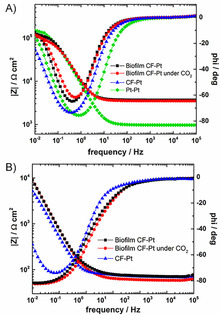
Bode plots of A) four different two‐electrode configurations including biofilm coated CF and Pt under N_2_‐saturated condition (▪), under CO_2_saturated condition (•), bare CF and Pt (▴), and Pt and Pt systems (⧫), and B) three different three‐electrode configurations including biofilm coated CF and Pt under N_2_‐saturated (▪), under CO_2_‐saturated condition (•), and CF and Pt systems (▴).

The electrochemical impedance experiments were performed in order to characterize the microbial electrochemical set‐up. The Bode plot for the two‐electrode systems is shown in Figure 8 A and all data is summarized in Table 2. Measured with the two‐electrode system (Pt−Pt), the resistance of the 0.2 m phosphate buffer solution pH 7.0 (*R*_sol_) was 85.0 Ω cm^−2^. Additionally, the resistance of the Nafion membrane (R_NF_) was determined as 0.87 kΩ cm^−2^. In the obtained electrical circuit, the resistance of the electrolyte and the Nafion membrane are connected in series with each other. The resistance of the working electrodes (R_WE_), which are the bare CF electrode and the one coated with the biofilm, were observed as 60.5 and 462.0 kΩ cm^−2^, respectively. The constant phase element (CPE) was used for the description of non‐ideal capacity of the sponge‐like CF electrode. Hence, the resistance of the biofilm was found to be 65.6 kΩ cm^−2^ and the capacitance of the CF coated with the biofilm of *M. extorquens* is calculated as 0.058 F cm^−2^. Based on the electrochemical impedance spectroscopy results, the two‐compartment configuration was characterized in detail and indicated negligible losses of the system. To characterize the whole electrochemical set‐up, an Ag/AgCl reference electrode was introduced into the cell. The Bode plot of the three‐electrode set‐up is shown in Figure 8 B and the corresponding data is summarized in Table 2. For the complete analysis of the electrochemical impedance spectroscopy, the IviumSoft developed by Ivium Technologies was applied as the evaluation program. Based on the fit of the electrochemical impedance spectra, the values listed in Table 2 were extracted. The electrochemical circuits used therefore, are provided in the Figure S8 in combination with the fitting plot. The corresponding Nyquist plots are also provided in the Supporting Information (Figure S9).

**Table 2:** List of impedance data for the two‐electrode and three‐electrode systems.

Condition	WE	*R*_sol_ / Ω cm^−2^	*R*_WE_ / kΩ cm^−2^	*R*_NF_ / kΩ cm^−2^	*R*_Bac_ / kΩ cm^−2^	*C*_Bac_ / F cm^−2^	CPE‐T	CPE‐P
**Two‐electrode system**								
0.2 m PB pH 7.0	Pt	85.0	159.4	0.87	–	–	0.0028	10.4
0.2 m PB pH 7.0	CF	317.5	462.0	3.2	–	–	0.023	38.2
0.2 m PB pH 7.0	biofilm CF	386.1	60.5	3.2	65.6	0.058	0.095	34.7
0.2 m PB pH 7.0 saturated with CO_2_	biofilm CF	364.6	81.9	3.2	30.5	0.55	0.035	39.1
**Three‐electrode system**								
0.2 m PB pH 7.0	CF	71.6	9.5	–	–	–	3.4	37.7
0.2 m PB pH 7.0	biofilm CF	74.5	263.5	–	38.3	2.1	2.7	40.1
0.2 m PB pH 7.0 saturated with CO_2_	biofilm CF	60.3	497.6	–	19.4	2.4	2.7	40.0

## Conclusions

We studied the electrocatalysis of biofilm consisting of *M. extorquens* for the electrochemical reduction of CO_2_ to formate. The formation of the biofilm on the sponge‐like carbon‐based electrode together with the continuous H_2_ production was observed during the biofilm growth period. As *M. extorquens* is known as a microorganism capable of reducing CO_2_ to formate as a part of its metabolism, we studied the utilization of this biofilm for the reduction of CO_2_ to formate by bio‐electrochemical catalysis, purging the system with CO_2_. The three microbial electrosynthesis cells were set up as described using CO_2_ as the only carbon source. By applying a constant potential of −0.75 V versus Ag/AgCl, the chromatographic analysis showed that formate was produced from the direct electron injection process from the carbon‐based electrode to the microorganisms. Additionally, the effects of neutral red as a redox mediator, were also investigated in the performance of the systems in homogeneous as well as heterogeneous way using an electropolymerized neutral red film on the electrode prior to the growing of the biofilm. The increase in the formate production rate and the Faradaic efficiency of the cells having neutral red is attributed to the improvement of the electron transfer from the electrode onto the biosystems. The heterogeneous approach using poly(neutral red) provides a simplification of the system as it reduces the contamination and product separation in the reaction chamber. Long‐term studies of up to 17 weeks demonstrated continuous formate formation, showing the stability and sustainability of the bio‐electrosynthesis systems. Further investigations are underway and we strongly propose these microbial electrosynthesis systems for renewable energy storage (chemical energy storage using CO_2_ conversion), CO_2_ reduction in general (CCU) and bio(electro)catalysis for other biotechnological conversions.

## Experimental Section

**Chemicals, materials and methods**: Carbon felt electrodes were purchased from SGL Carbon GmbH and a Pt wire was used for the electrical contact. Nafion perfluorinated membrane (Nafion 324) was purchased from Sigma–Aldrich. All chemicals were of analytical grade, purchased from commercial sources and used without further purification. The phosphate buffer (PB) solutions were prepared from appropriate amounts of K_2_HPO_4_ and KH_2_PO_4_ solution to reach desired pH value.

The glass substrates were cleaning via sonication for 15 mins each in acetone, 2 % Hellmanex solution, deionized water and 2‐propanol at the last step. Then, the glass substrates were treated in an evaporation chamber for thermal evaporation of 5 nm chromium followed by 80 nm gold as being the Cr/Au substrate for FTIR measurement. A transparent Cr/Au‐coated glass electrode was prepared in a similar manner with thickness of 3 nm chromium and 15 nm gold for UV/Vis measurement., the Cr/Au electrode was used with the thickness of 5 nm of chromium and 80 nm of gold. For UV/Vis measurement, the transparent Cr/Au electrode was used with the thickness of 3 nm of chromium and 15 nm of gold. The absorption spectra were recorded on a PerkinElmer Lambda 1050 UV/Vis/NIR spectrophotometer.

ATR‐FTIR spectra of neutral red and poly(neutral red) were recorded on a Bruker Vertex 80‐ATR machine over 128 scans. The absorption spectra of neutral red aqueous solution and films were measured at room temperature on a PerkinElmer Lambda 1050 UV/Vis/NIR spectrophotometer. The monomer film was prepared by drop‐casting aqueous solution of neutral red at the concentration of 0.5 mg mL^−1^ on a transparent Cr/Au electrode.

SEM images were obtained using JEOL JSM‐6360 LV scanning electron microscope at the accelerating voltage of 7.0 kV.

**Methylobacterium extorquens pure culture**: *M. extorquens* DSM‐1337 was obtained from the Deutsche Sammlung Mikroorganismen und Zellkulturen (DSMZ). The medium used for cultivation containing polypeptone (10 g L^−1^), yeast extract (2 g L^−1^), and MgSO_4_⋅7 H_2_O (2 g L^−1^) and supplemented with 0.5 % (*w*/*v*) methanol. Na_2_WO_4_ and Na_2_MoO_4_ were added separately to the medium to a final concentration of 0.3 μm each. The pH was adjusted to a value of 7.0 after autoclaving. *M. extorquens* cultures were routinely grown in an incubator at 30 °C at 100 rpm.

**Electrochemical studies**: All electrochemical experiments were carried out using an IVIUM CompactStat (The Netherlands) instrument and the potential values reported in this work referred to Ag/AgCl (3 m KCl).

**Preparation of poly(neutral red) coated on a carbon felt electrode (PNR/CF)**

**Set‐up**: The experiments were performed in a one‐compartment electrochemical cell with three‐electrode system. A CF (2.5×5.6×0.6 cm^3^) electrode, a Ag/AgCl (3 m KCl) electrode and a Pt plate (1.4×4.1 cm^2^) were used as a working electrode, a reference electrode and a counter electrode, respectively.

**Electrochemical polymerization of neutral red**: Following the previous report,^55^ before use, the CF electrode was electrochemically pre‐treated in 0.1 m KNO_3_ aqueous solution at potentials between 0 and 1.0 V with the scan rate of 50 mV s^−1^ over 20 cycles. After that, the electropolymerization of neutral red was carried out at potentials between −1.0 and 1.0 V with the scan rate of 50 mV s^−1^ over 20 cycles in 0.1 m phosphate buffer solution pH 6.0 containing 1 mm neutral red and 0.1 m KNO_3_, yielding a poly(neutral red) coated CF (PNR/CF). The PNR/CF electrode was electrochemically characterized at potentials between −1.0 and 1.0 V at different scan rates of 5, 10, 25, 50 and 100 mV s^−1^. For FTIR and UV/Vis measurements, the poly(neutral red) was electropolymerized onto Cr/Au‐coated glass electrodes with the similar manner to the CF electrode.

**Microbial electrosynthesis studies**

*Set‐up and electrolytes*: The inoculation and microbial electrosynthesis experiments were carried out in the two‐compartment electrochemical cell of which the anode and cathode compartments were separated by a pretreated Nafion membrane, allowing proton transport in the system. The membrane was prepared by soaking a commercial Nafion 324 sheet in a 3 m HCl solution for 2 h followed by boiling the membrane in deionized water for an additional 30 min. The three‐electrode cell consists of CF or PNR/CF (2.5×6.5×0.6 cm^3^) electrode as a working electrode, a Ag/AgCl (3 m KCl) reference electrode and a Pt plate (1.4×4.1 cm^2^) counter electrode. The anode compartment contained 0.2 m phosphate buffer solution pH 7.0 as a supporting electrolyte solution. For the biofilm growth phase, the cathodic electrolyte was a medium consisting of the following nutrients: polypeptone (10 g L^−1^), yeast extract (2 g L^−1^), MgSO_4_⋅7 H_2_O (2 g L^−1^), methanol (0.5 % *w*/*v*) and Na_2_WO_4_ (0.3 μm).^51^ During the microbial electrosynthesis studies, 0.2 m phosphate buffer solution pH 7.0 was used as cathodic electrolyte solution.

*Inoculation of microorganisms*: The formation of biofilm on CF electrodes was done in the aforementioned electrochemical set‐up in which the cathodic electrolyte solution contained 10 % (*v*/*v*) of *M. extorquens* pure culture and afterwards purged with CO_2_ for 2 h. A constant potential at −0.75 V was applied for a month with weekly CO_2_ purging. During this period, 2 mL of headspace samples were collected daily and injected into a gas chromatography (GC) for the analysis of headspace products (CH_4_, CO and H_2_). After one‐month inoculation of the *M. extorquens*, the biofilm formation on the electrode could be observed. The medium was removed and replaced one or two times with 0.2 m phosphate buffer solution pH 7.0 in order to get rid of the remaining media that went inside the sponge‐like CF electrodes.

*Long‐term microbial electrosynthesis studies*: In this work, 3 different microbial electrosynthesis cells (MEC 1, 2 and 3) were investigated. For all MECs, the long‐term electrolysis was conducted constantly at applied potential of −0.75 V under CO_2_‐saturated condition. The cathodic chambers were purged weekly with CO_2_ for 2 h in order to keep the systems saturated with CO_2_, and the electrolyte was replaced with new buffer solutions every 3–4 weeks reported as different running cycles. The experiments were continuously performed for 8, 12 and 17 weeks for MEC 1, MEC 2 and MEC 3, respectively. In case of MEC 2, neutral red solution was added into the cathodic electrolyte solution to achieve a final concentration of 50 μm.

*Product analysis*: The headspace samples were taken using a gas‐tight syringe and the product analysis for H_2_ and CO was done by using a Thermo Scientific Trace GC Ultra equipped with a thermal conductivity detector (TCD). For the formate production analysis, the liquid samples were diluted with deionized water and their concentrations were determined by a Thermo Scientific Dionex‐5000 ion chromatography (IC) system equipped with an IonPac AG19 guard column (2×50 mm), a Dionex AS19 column (2×250 mm) and a Dionex suppressor‐conductivity detector by using gradient concentration of KOH as eluent. The Faradaic efficiency (%FE) toward the product is calculated as:

$$\%FE=\;\frac{moles\;of\;product}{\frac{1}{n}\times moles\;of\;electron}\;\times 100$$

in which moles of product are calculated from the amount of H_2_ or formate produced in the systems, *n* is the number of electrons needed for reduction (in both H_2_ and formate production cases, *n*=2) and moles of electron are calculated by the dividing of number of charges during electrolysis by Faradaic constant as 96 485.33 C⋅mol^−1^.

**Electrochemical characterization**

*Characterization by cyclic voltammetry (CV)*: The biofilm electrodes were characterized electrochemically after inoculation period, by means of cyclic voltammetry under the N_2_‐ and CO_2_‐saturated conditions for all MECs. CVs were recorded in 0.2 m phosphate buffer solution at pH 7.0 (with 50 μm neutral red for MEC 2) at potentials between 0 and −850 mV vs. Ag/AgCl (3 m KCl) with the scan rate of 1 mV s^−1^ by using a Jaissle Potentiostat–Galvanostat IMP 88 PC.

*Characterization by electrochemical impedance spectroscopy (EIS)*: All impedance experiments were carried out in a two‐compartment electrochemical cell separated with a pretreated Nafion membrane. The impedance spectra were recorded in 0.2 m potassium buffer solution pH 7.0 within the frequency range of 10^5^ to 10^−2^ Hz at the perturbation amplitude of 50 mV. In order to determine the parameters of the cell, two Pt plates were used as electrodes in the abovementioned set‐up as a control experiment. Next, one Pt electrode was replaced by a CF and used as a working electrode. Later on, the CF was replaced by the CF coated with the biofilm. Additionally, a Ag/AgCl (3 m KCl) was introduced to the set‐up to achieve the three‐electrode, in order to characterize the complete electrochemical cell by the impedance spectroscopy. The vindication for this procedure is explained in more detail in a separate publication.^61^

## Conflict of interest

*The authors declare no conflict of interest*.

## Supporting information

As a service to our authors and readers, this journal provides supporting information supplied by the authors. Such materials are peer reviewed and may be re‐organized for online delivery, but are not copy‐edited or typeset. Technical support issues arising from supporting information (other than missing files) should be addressed to the authors.

Supplementary

## References

[1] A. P. M. Baede , P. van der Linden , A. Verbruggen , Annex II, to IPCC Fourth Assessment Report 2007.

[2] D. Lüthi , M. Le Floch , B. Bereiter , T. Blunier , J.-M. Barnola , U. Siegenthaler , D. Raynaud , J. Jouzel , H. Fischer , K. Kawamura , T. F. Stocker , Nature 2008, 453, 379–382. 18480821 10.1038/nature06949

[3] S. Arrhenius , Phil. Mag. S. 5 1896, 41, 237–276.

[4] M. Aresta , A. Dibenedetto , in CO_2_ Conversion and Utilization, American Chemical Society, 2002, pp. 54–70.

[5] M. Aresta , A. Dibenedetto , Dalton Trans. 2007, 0, 2975. 10.1039/b700658f 17622414

[6] E. V. Kondratenko , G. Mul , J. Baltrusaitis , G. O. Larrazábal , J. Pérez-Ramírez , Energy Environ. Sci. 2013, 6, 3112.

[7] K. Schuchmann , V. Müller , Science 2013, 342, 1382–1385. 24337298 10.1126/science.1244758

[8] K. B. Cantrell , T. Ducey , K. S. Ro , P. G. Hunt , Bioresour. Technol. 2008, 99, 7941–7953. 18485701 10.1016/j.biortech.2008.02.061

[9] S.-Y. Chiu , C.-Y. Kao , C.-H. Chen , T.-C. Kuan , S.-C. Ong , C.-S. Lin , Bioresour. Technol. 2008, 99, 3389–3396. 17904359 10.1016/j.biortech.2007.08.013

[10] D. T. Whipple , P. J. A. Kenis , J. Phys. Chem. Lett. 2010, 1, 3451–3458.

[11] A. A. Peterson , J. K. Nørskov , J. Phys. Chem. Lett. 2012, 3, 251–258.

[12] Y. Hori , K. Kikuchi , S. Suzuki , Chem. Lett. 1985, 14, 1695–1698.

[13] K. P. Kuhl , E. R. Cave , D. N. Abram , T. F. Jaramillo , Energy Environ. Sci. 2012, 5, 7050.

[14] T. Inoue , A. Fujishima , S. Konishi , K. Honda , Nature 1979, 277, 637–638.

[15] C. Wang , Z. Xie , K. E. deKrafft , W. Lin , J. Am. Chem. Soc. 2011, 133, 13445–13454. 21780787 10.1021/ja203564w

[16] B. Kumar , M. Llorente , J. Froehlich , T. Dang , A. Sathrum , C. P. Kubiak , Annu. Rev. Phys. Chem. 2012, 63, 541–569. 22404587 10.1146/annurev-physchem-032511-143759

[17] J. Hawecker , J.-M. Lehn , R. Ziessel , Helv. Chim. Acta 1986, 69, 1990–2012.

[18] J. M. Lehn , R. Ziessel , Proc. Natl. Acad. Sci. USA 1982, 79, 701–704. 16593151 10.1073/pnas.79.2.701 PMC345815

[19] H. A. Hansen , J. B. Varley , A. A. Peterson , J. K. Nørskov , J. Phys. Chem. Lett. 2013, 4, 388–392. 26281729 10.1021/jz3021155

[20] J. Shen , R. Kortlever , R. Kas , Y. Y. Birdja , O. Diaz-Morales , Y. Kwon , I. Ledezma-Yanez , K. J. P. Schouten , G. Mul , M. T. M. Koper , Nat. Commun. 2015, 6, 8177. 26324108 10.1038/ncomms9177 PMC4569799

[21] C. Costentin , S. Drouet , M. Robert , J.-M. Savéant , Science 2012, 338, 90–94. 23042890 10.1126/science.1224581

[22] M. Asadi , B. Kumar , A. Behranginia , B. A. Rosen , A. Baskin , N. Repnin , D. Pisasale , P. Phillips , W. Zhu , R. Haasch , R. F. Klie , P. Král , J. Abiade , A. Salehi-Khojin , Nat. Commun. 2014, 5, 4470. 25073814 10.1038/ncomms5470

[23] S. Lin , C. S. Diercks , Y.-B. Zhang , N. Kornienko , E. M. Nichols , Y. Zhao , A. R. Paris , D. Kim , P. Yang , O. M. Yaghi , C. J. Chang , Science 2015, 349, 1208–1213. 26292706 10.1126/science.aac8343

[24] R. Kortlever , I. Peters , S. Koper , M. T. M. Koper , ACS Catal. 2015, 5, 3916–3923.

[25] Y. Chen , C. W. Li , M. W. Kanan , J. Am. Chem. Soc. 2012, 134, 19969–19972. 23171134 10.1021/ja309317u

[26] C. W. Li , M. W. Kanan , J. Am. Chem. Soc. 2012, 134, 7231–7234. 22506621 10.1021/ja3010978

[27] Y. Hori , H. Wakebe , T. Tsukamoto , O. Koga , Electrochim. Acta 1994, 39, 1833–1839.

[28] A. M. Appel , J. E. Bercaw , A. B. Bocarsly , H. Dobbek , D. L. DuBois , M. Dupuis , J. G. Ferry , E. Fujita , R. Hille , P. J. A. Kenis , C. A. Kerfeld , R. H. Morris , C. H. Peden , A. R. Portis , S. W. Ragsdale , T. B. Rauchfuss , J. N. Reek , L. C. Seefeldt , R. K. Thauer , G. L. Waldrop , Chem. Rev. 2013, 113, 6621–6658. 23767781 10.1021/cr300463y PMC3895110

[29] M. Aresta , A. Dibenedetto , C. Pastore , Environ. Chem. Lett. 2005, 3, 113–117.

[30] S. Schlager , A. Fuchsbauer , M. Haberbauer , H. Neugebauer , N. S. Sariciftci , J. Mater. Chem. A 2017, 5, 2429–2443.

[31] N. Long , J. Lee , K.-K. Koo , P. Luis , M. Lee , Energies 2017, 10, 473.

[32] S. Schlager , A. Dibenedetto , M. Aresta , D. H. Apaydin , L. M. Dumitru , H. Neugebauer , N. S. Sariciftci , Energy Technol. 2017, 5, 812–821. 10.1002/ente.201600610 PMC5488624 28748135

[33] J. Shi , Y. Jiang , Z. Jiang , X. Wang , X. Wang , S. Zhang , P. Han , C. Yang , Chem. Soc. Rev. 2015, 44, 5981–6000. 26055659 10.1039/c5cs00182j

[34] J. R. Andreesen , L. G. Ljungdahl , J. Bacteriol. 1973, 116, 867–873. 4147651 10.1128/jb.116.2.867-873.1973 PMC285457

[35] K. Seelbach , B. Riebel , W. Hummel , M.-R. Kula , V. I. Tishkov , A. M. Egorov , C. Wandrey , U. Kragl , Tetrahedron Lett. 1996, 37, 1377–1380.

[36] S. Schlager , H. Neugebauer , M. Haberbauer , G. Hinterberger , N. S. Sariciftci , ChemCatChem 2015, 7, 967–971. 26113881 10.1002/cctc.201402932 PMC4471636

[37] S. Schlager , L. M. Dumitru , M. Haberbauer , A. Fuchsbauer , H. Neugebauer , D. Hiemetsberger , A. Wagner , E. Portenkirchner , N. S. Sariciftci , ChemSusChem 2016, 9, 631–635. 26890322 10.1002/cssc.201501496 PMC5067720

[38] T. Reda , C. M. Plugge , N. J. Abram , J. Hirst , Proc. Natl. Acad. Sci. USA 2008, 105, 10654–10658. 18667702 10.1073/pnas.0801290105 PMC2491486

[39] B. A. Parkinson , P. F. Weaver , Nature 1984, 309, 148–149.

[40] B. E. Logan , K. Rabaey , Science 2012, 337, 686–690. 22879507 10.1126/science.1217412

[41] A. Alissandratos , H.-K. Kim , H. Matthews , J. E. Hennessy , A. Philbrook , C. J. Easton , Appl. Environ. Microbiol. 2013, 79, 741–744. 23144135 10.1128/AEM.02886-12 PMC3553769

[42] S. Cheng , D. Xing , D. F. Call , B. E. Logan , Environ. Sci. Technol. 2009, 43, 3953–3958. 19544913 10.1021/es803531g

[43] M. C. A. A. Van Eerten-Jansen , A. Ter Heijne , C. J. N. Buisman , H. V. M. Hamelers , Int. J. Energy Res. 2012, 36, 809–819.

[44] M. Villano , F. Aulenta , C. Ciucci , T. Ferri , A. Giuliano , M. Majone , Bioresour. Technol. 2010, 101, 3085–3090. 20074943 10.1016/j.biortech.2009.12.077

[45] L. G. Ljungdhal , Annu. Rev. Microbiol. 1986, 40, 415–450. 3096193 10.1146/annurev.mi.40.100186.002215

[46] K. T. Wieringa , Antonie Van Leeuwenhoek 1936, 3, 263–273.

[47] K. T. Wieringa , Antonie Van Leeuwenhoek 1939, 6, 251–262.

[48] S. W. Ragsdale , E. Pierce , Biochim. Biophys. Acta-Proteins Proteomics 2008, 1784, 1873–1898. 10.1016/j.bbapap.2008.08.012 PMC2646786 18801467

[49] J. Schrader , M. Schilling , D. Holtmann , D. Sell , M. V. Filho , A. Marx , J. A. Vorholt , Trends Biotechnol. 2009, 27, 107–115. 19111927 10.1016/j.tibtech.2008.10.009

[50] S. Belkhelfa , K. Labadie , C. Cruaud , J.-M. Aury , D. Roche , M. Bouzon , M. Salanoubat , V. Döring , Genome Announc. 2018, 6, e00018-18. 10.1128/genomeA.00018-18 PMC5824006 29472323

[51] H. Hwang , Y. J. Yeon , S. Lee , H. Choe , M. G. Jang , D. H. Cho , S. Park , Y. H. Kim , Bioresour. Technol. 2015, 185, 35–39. 25746476 10.1016/j.biortech.2015.02.086

[52] T. D. Harrington , V. N. Tran , A. Mohamed , R. Renslow , S. Biria , L. Orfe , D. R. Call , H. Beyenal , Bioresour. Technol. 2015, 192, 689–695. 26094195 10.1016/j.biortech.2015.06.037 PMC4516386

[53] D. H. Park , M. Laivenieks , M. V. Guettler , M. K. Jain , J. G. Zeikus , Appl. Environ. Microbiol. 1999, 65, 2912–2917. 10388683 10.1128/aem.65.7.2912-2917.1999 PMC91436

[54] D. H. Park , J. G. Zeikus , Appl. Environ. Microbiol. 2000, 66, 1292–1297. 10742202 10.1128/aem.66.4.1292-1297.2000 PMC91983

[55] R. Pauliukaite , M. E. Ghica , M. Barsan , C. M. A. Brett , J. Solid State Electrochem. 2007, 11, 899–908.

[56] D. R. Shobha Jeykumari , S. Sriman Narayanan , Biosens. Bioelectron. 2008, 23, 1404–1411. 18294834 10.1016/j.bios.2007.12.007

[57] A. A. Karyakin , O. A. Bobrova , E. E. Karyakina , J. Electroanal. Chem. 1995, 399, 179–184.

[58] Y. Ren , D. Pan , X. Li , F. Fu , Y. Zhao , X. Wang , J. Chem. Technol. Biotechnol. 2013, 88, 1946–1950.

[59] C. Li , L. Ding , H. Cui , L. Zhang , K. Xu , H. Ren , Bioresour. Technol. 2012, 116, 459–465. 22534369 10.1016/j.biortech.2012.03.115

[60] Y. Zou , C. Xiang , L. Yang , L.-X. Sun , F. Xu , Z. Cao , Int. J. Hydrogen Energy 2008, 33, 4856–4862.

[61] H. Coskun , A. Aljabour , P. De Luna , D. Farka , T. Greunz , D. Stifter , M. Kus , X. Zheng , M. Liu , A. W. Hassel , W. Schöfberger , E. H. Sargent , N. S. Sariciftci , S. Philipp , Sci. Adv. 2017, 3, e1700686. 10.1126/sciadv.1700686 PMC5544399 28798958

[62] V. Anesti , J. Vohra , S. Goonetilleka , I. R. McDonald , B. Straubler , E. Stackebrandt , D. P. Kelly , A. P. Wood , Environ. Microbiol. 2004, 6, 820–830. 15250884 10.1111/j.1462-2920.2004.00623.x

[63] M. E. Lidstrom , L. Chistoserdova , J. Bacteriol. 2002, 184, 1818. 11889085 10.1128/JB.184.7.1818.2002 PMC134909

[64] V. Müller , Appl. Environ. Microbiol. 2003, 69, 6345–6353. 14602585 10.1128/AEM.69.11.6345-6353.2003 PMC262307

[65] D. Das , T. N. Veziroǧlu , Int. J. Hydrogen Energy 2001, 26, 13–28.

[66] C. Yang , J. Yi , X. Tang , G. Zhou , Y. Zeng , React. Funct. Polym. 2006, 66, 1336–1341.

[67] A. Terada , A. Yuasa , T. Kushimoto , S. Tsuneda , A. Katakai , M. Tamada , Microbiology 2006, 152, 3575–3583. 17159210 10.1099/mic.0.28881-0

[68] T. Saito , M. Mehanna , X. Wang , R. D. Cusick , Y. Feng , M. A. Hickner , Bioresour. Technol. 2011, 102, 395–398. 20889061 10.1016/j.biortech.2010.05.063

[69] S. Cheng , B. E. Logan , Electrochem. commun. 2007, 9, 492–496.

