# Supplementary material for: Enhanced Bio‐Electrochemical Reduction of Carbon Dioxide by Using Neutral Red as a Redox Mediator
Source: Chembiochem. 2019 Mar 12;20(9):1196–205. doi: 10.1002/cbic.201800784 (PMC9328444; doi:10.1002/cbic.201800784)
Supplement: Supplementary file 1 — Supplementary [file CBIC-20-1196-s001.pdf]

CHEMBIOCHEM

Supporting Information

Enhanced Bio-Electrochemical Reduction of Carbon Dioxide by Using Neutral Red as a Redox Mediator

Hathaichanok Seelajaroen,*[a] Marianne Haberbauer,[b] Christine Hemmelmair,[b] Abdalaziz Aljabour,[c] Liviu Mihai Dumitru,*[a] Achim Walter Hassel,[c] and Niyazi Serdar Sariciftci[a]

cbic_201800784_sm_miscellaneous_information.pdf

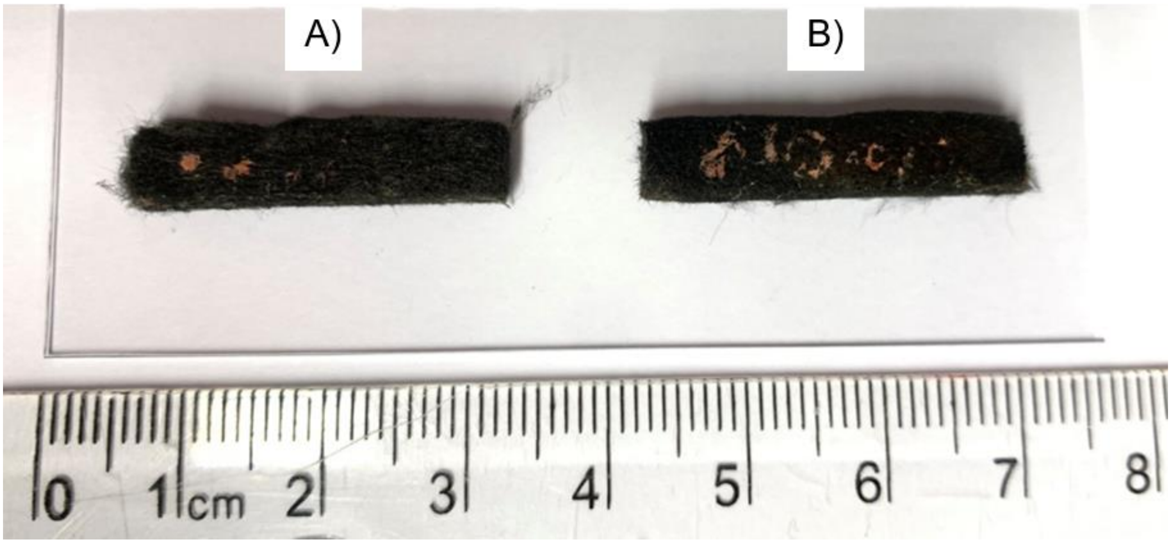

Figure S1. Photograph of dried A) CF with biofilm and B) PNR/CF with biofilm.

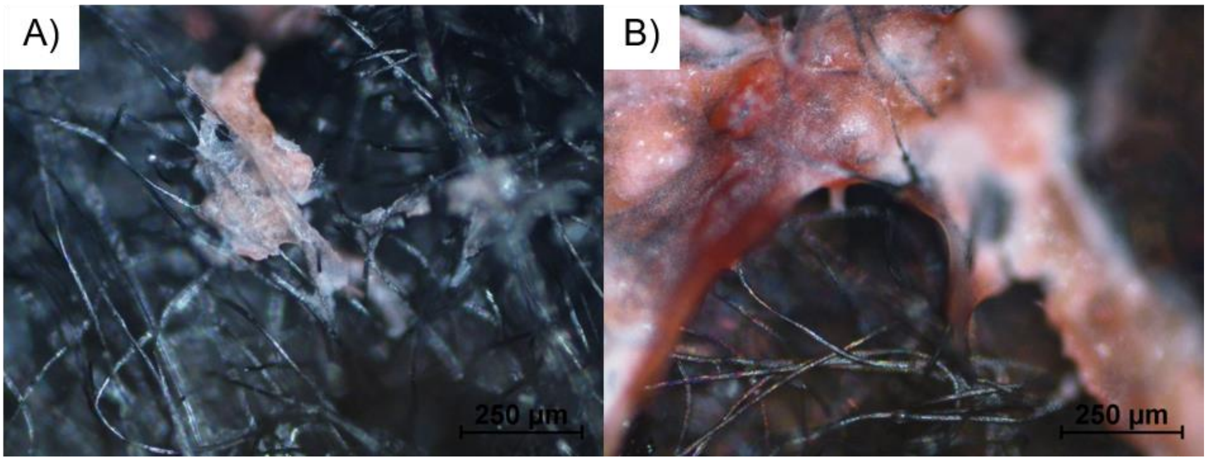

Figure S2. Optical microscopic images of A) CF with biofilm and B) PNR/CF with biofilm. Bar, 250 µm.

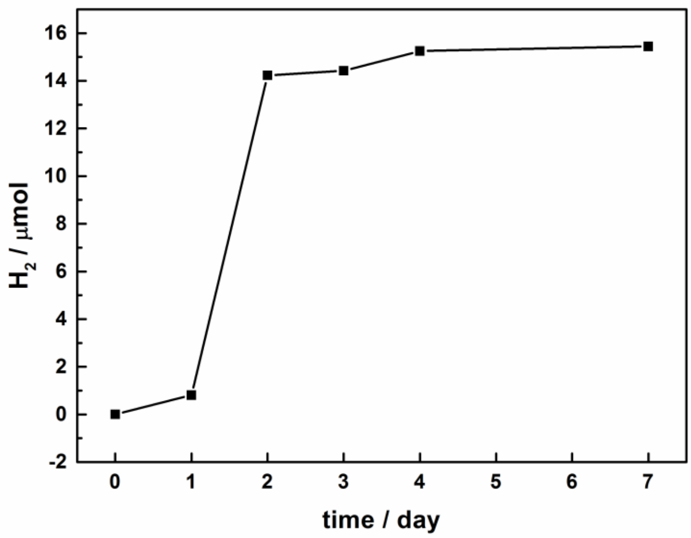

Figure S3. Amount of H_2 observed in the control experiment of a bare CF performed in the growing medium under N_2-saturated condition.

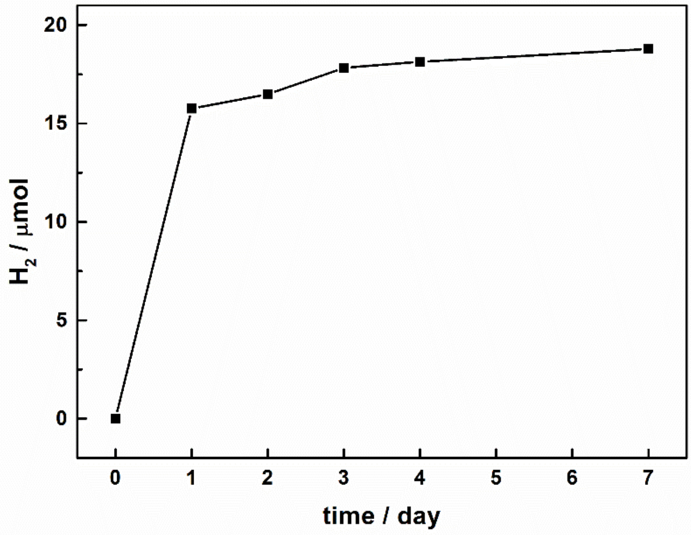

Figure S4. Amount of H_2 observed in the control experiment of a bare CF performed in the growing medium under CO_2-saturated condition.

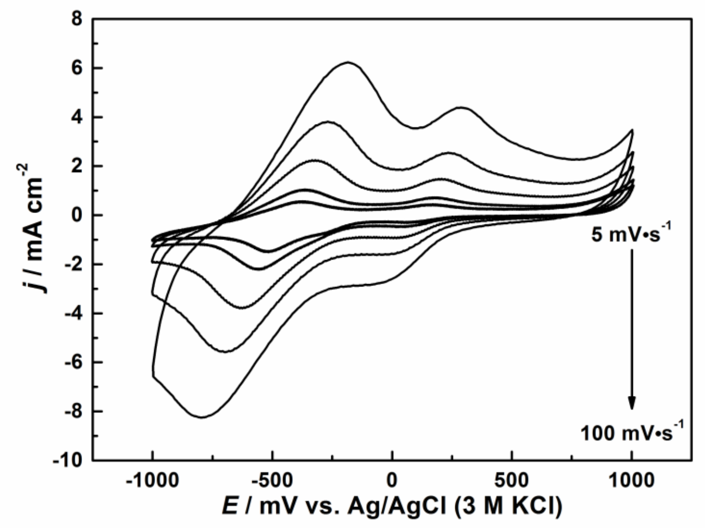

Figure S5. Cyclic voltammograms of poly(neutral red) in 0.1 M phosphate buffer solution pH 6.0 at different scan rates (5, 10, 25, 50 and 100 mV·s^{-1}).

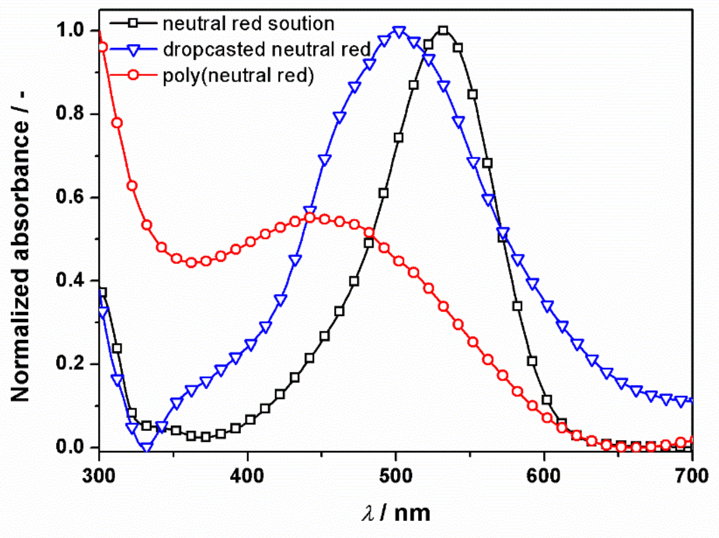

Figure S6. UV/Vis spectra of neutral aqueous solution (black line), drop-casted neutral red on a transparent Cr/Au substrate and poly(neutral red) coated on a transparent Cr/Au substrate.

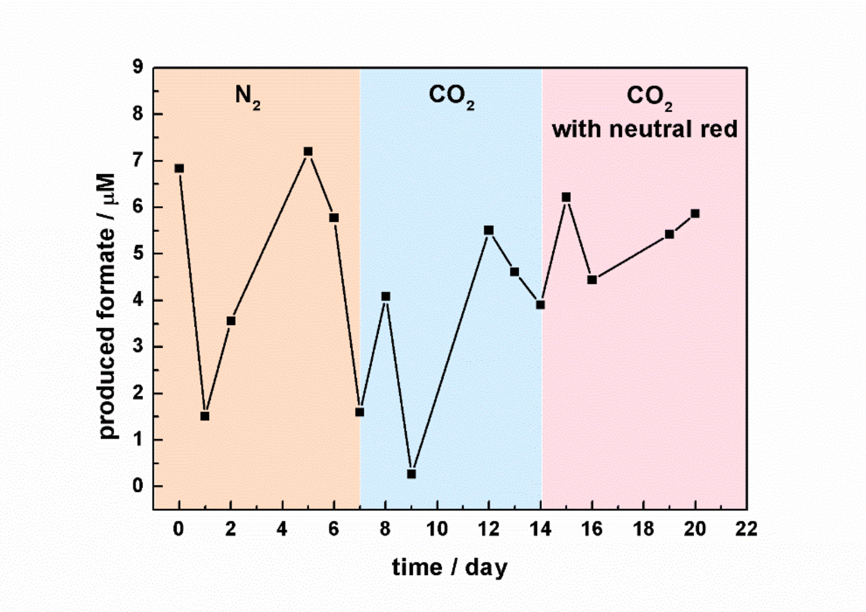

Figure S7. Concentration of formate ion observed in the set of control experiments of a bare CF in 0.2 M phosphate buffer solution pH 7.0 under N_2-saturated atmosphere (day 0 to day 7), under CO_2-saturated atmosphere (day 8 to day 14) and under CO_2-saturated atmosphere containing 50 μM of neutral red (day 15 to day 20).

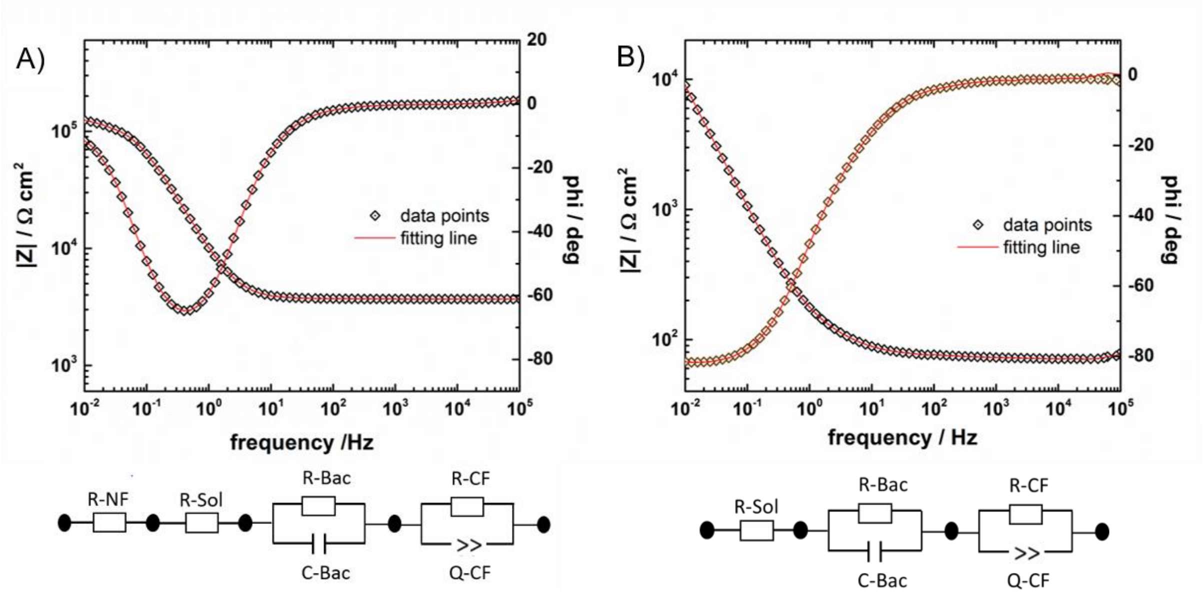

Figure S8. Bode plots (top) and equivalent circuits (below) used for data analysis of A) two-electrode configuration and B) three-electrode configuration of a a biofilm coated CF and Pt showing data points as black square points and fitting curve as red line.

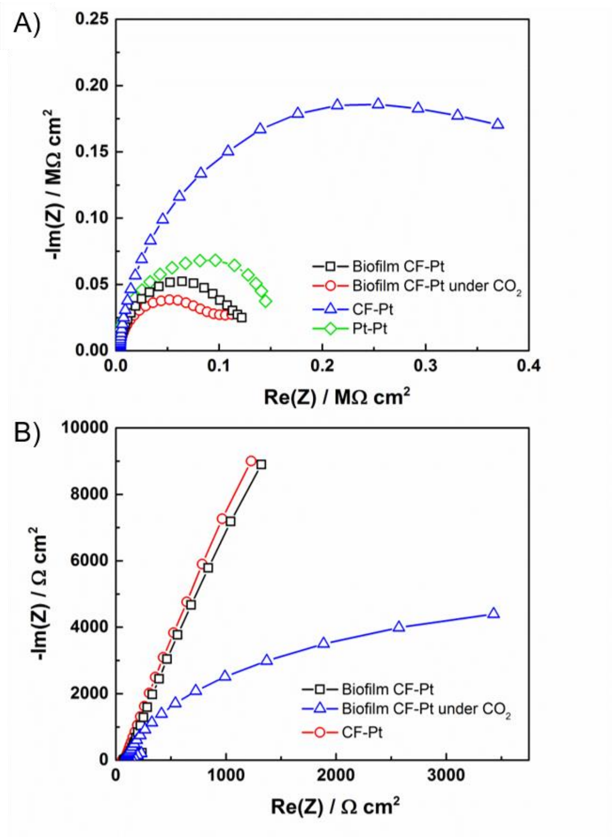

Figure S9. Nyquist plots of A) four different two-electrode configurations including biofilm coated CF and Pt under N_2-saturated condition (black line), under CO_2-saturated condition (red line), bare CF and Pt (blue line), and Pt and Pt systems (green line), and B) three different three-electrode configurations including biofilm coated CF and Pt under N_2-saturated (black line), under CO_2-saturated condition (red line), and CF and Pt systems (blue line).
